# A 42-year-old woman with abnormal uterine bleeding—leiomyoma (AUB-L) reporting a hemoglobin of 1.6 g/dL: a case report

**DOI:** 10.1186/s13256-024-04593-1

**Published:** 2024-06-20

**Authors:** Shamsa M. Qaadri, Tavsimran S. Luthra, Kumarie Budhu, Or Sagi

**Affiliations:** 1 St. George’s University School of Medicine, West Indies, Grenada; 2 Department of Obstetrics & Gynecology, The Brooklyn Hospital Center, New York, USA

**Keywords:** Abnormal uterine bleeding, Anemia, Low hemoglobin, Leiomyoma, Case report

## Abstract

**Background:**

Abnormal uterine bleeding, formerly known as menometrorrhagia, is estimated to occur in up to one-third of women, commonly at menarche or perimenopause. Among many other causes, abnormal uterine bleeding is known to be caused by leiomyomas, and is itself a leading cause of severe iron deficiency and iron deficiency anemia in women. Rarely, abnormal uterine bleeding can lead to critically low hemoglobin values of less than 2 g/dL. We report here a case of a woman with abnormal uterine bleeding caused by leiomyomas presenting with severely low hemoglobin.

**Case presentation:**

We report the case of a 42-year-old Asian American woman who presented to the emergency department with chronic abnormal uterine bleeding and symptoms of anemia, including multiple syncopal episodes and abnormally pale skin but otherwise alert and oriented. Laboratory tests found a record-low hemoglobin of 1.6 g/dL and hematocrit of 6%. Transabdominal pelvic ultrasound revealed a lower uterine segment/cervical fibroid measuring 7.5 × 5 × 7.8 cm (length × depth × width). Patient was diagnosed with abnormal uterine bleeding–leiomyoma and received five units of packed red blood cells, one unit of fresh frozen plasma, Venofer infusions, tranexamic acid, and medroxyprogesterone. She was discharged from the hospital after 4 days.

**Conclusion:**

To date, only a handful of cases have been reported of female patient survival following severely low hemoglobin caused by abnormal uterine bleeding. This case adds to this literature, highlighting the remarkable degree of compensation that can lead to an alert, ambulatory, and oriented patient with abnormal uterine bleeding caused by leiomyoma.

## Background

Abnormal uterine bleeding (AUB) is a broad term that refers to bleeding from the uterus that is longer than usual or that occurs irregularly. While the normal menstrual cycle has a frequency of 24–38 days and typically lasts 2–7 days with 5–80 mL of blood loss, variations from any of these features may constitute AUB.

Etiology of abnormal uterine bleeding is complex. Many of the factors that disrupt menstruation can cause AUB. These include structural alterations such as leiomyoma, polyps, adenomyosis, hyperplasia, or disruptions in clotting pathways and the hypothalamic–pituitary–ovarian axis [1]. Importantly, AUB is a symptom not a diagnosis, and its underlying causes must be investigated through laboratory and imaging testing.

On rare occasions, prolonged cases of acute AUB can lead to severe hemodilution and iron deficiency anemia [2]. We report herein a case of a 42-year-old Asian American woman with AUB caused by leiomyomas, causing a critically low hemoglobin of 1.6 g/dL.

## Case presentation

A 42-year-old Asian American woman, G1P0010, presented to the emergency department (ED) with chronic vaginal bleeding with a history of palpitation, weakness, light-headedness, and several syncopal episodes over the past 4 years.

On physical examination, the patient appeared alert and oriented to time, place, and person. Her skin appearance was remarkably pale. Her vitals upon admission were: temperature 98.1 °F (36.7 °C); heart rate 72 beats per minute; blood pressure (BP) 126/76; respiratory rate (RR) 22 on 100% oxygen. She denied smoking, drinking alcohol, or the use of any illicit substances. There were no focal neurological deficits. While normal S1 and S2 sounds were observed, there was a systolic murmur.

She had no history of sickle cell or other hematopoietic disorders. Previous obstetric history includes a spontaneous abortion more than 10 years ago. The patient reported a history of chronic, intermittent, heavy vaginal bleeding for the past 4 years that were self-managed. Severe bleeding in the past 2 months, including the passage of coin-sized blood clots, prompted a visit to the emergency room (ER). Presenting symptoms included continuous vaginal bleeding associated with symptoms of anemia, including exertional shortness of breath, syncope, palpitations, and dizziness.

### Investigations

Initial laboratory data on admittance is summarized in Table 1. Her hemoglobin (Hgb) level was critically low at 1.6 g/dL on arrival, with a low mean corpuscular volume (MCV) of 77 fL. Hematocrit was also critically low at 6%. Red blood cell distribution width (RDW) was elevated to 24%. Absolute reticulocyte count was elevated to 4.2%. Iron panel results were consistent with severe Iron Deficiency Anemia (IDA), with an iron level of 10 μg/dL and a total iron binding capacity of 464 μg/dL. Vitamin B12 was within the normal at 746 pg/mL, as well as platelet count at 316 K/cmm, Activated Partial Thromboplastin Time (aPTT) at 23.1 s (23.9–30.7 s), Prothrombin Time (PT) at 11.9 s (9.5–12.1 s), and International Normalized Ratio (INR) at 1.1 (0.9–1.1).

**Table 1:** Hematopoietic studies

Hematopoietic studies	Value	Reference range
Hemoglobin (g/dL)	1.6	11.5–14.1
Hematocrit (%)	6	35–42
Mean corpuscular volume (fL)	77	79–96
Mean corpuscular hemoglobin (pg)	22	27–31
Mean corpuscular hemoglobin concentration (g/dL)	28.5	32.0–36.0
Iron (μg/dL)	10	50–170
Total iron binding capacity (μg/dL)	464	179–378

The results for hemoglobin and hematocrit were verified by the laboratory.

A transabdominal pelvic ultrasound was ordered, which revealed the expansion of the endometrial cavity at the level of the lower uterine segment and upper cervix due to a large well-circumscribed heterogeneous mass without frank internal vascularity. The size of the submucosal pedunculated intracavitary fibroid was 7.5 × 5 × 7.8 cm (length × depth × width). Transvaginal ultrasound or hysteroscopy were not performed due to risk of rupturing the fibroid and the severe pain experienced during gynecologic exam.

### Diagnostic criteria

Previously used terms such as heavy menstrual bleeding, oligomenorrhea, menorrhagia, menometrorrhagia, and dysfunctional uterine bleeding have been discarded following new guidelines published in 2007, 2011, and 2018 by the International Federation of Obstetrics and Gynecology (FIGO) [3]. These revisions favor the use of simple addendums to describe the nature of abnormal uterine bleeding during reproductive years. The various subtypes of abnormal uterine bleeding identified by FIGO, including those related to uterine structural abnormalities and those unrelated, are summarized in Table 2.

**Table 2:** Summary of the underlying etiologies of abnormal uterine bleeding

P	Polyp
A	Adenomyosis
L	Leiomyoma
M	Malignancy and hyperplasia
C	Coagulopathy
O	Ovulatory dysfunction
E	Endometrial disorders
I	Iatrogenic
N	Not otherwise classified

Table 2. Summary of the underlying etiologies of abnormal uterine bleeding.

Utilizing the above criteria, the patient was diagnosed with severe symptomatic anemia secondary to abnormal uterine bleeding–leiomyoma (AUB-L).

### Treatment

Several treatment options are indicated with high efficacy in abnormal uterine bleeding, including coagulation agents (for example, tranexamic acid); oral contraceptives; oral progestins; endometrial ablation; progestin-releasing devices (for example, levonorgestrel-releasing intrauterine system); gonadotropin-releasing hormone (GnRH) agonists; and surgical intervention (for example, myomectomy and hysterectomy) [4]. Hemodynamic stability is achieved through packed red blood cells (pRBC) and fresh frozen plasma (FFP) transfusion.

The patient was given a 1000 mg solution of tranexamic acid via intravenous piggyback every 8 hours and oral tablets of 20 mg medroxyprogesterone every 8 hours. She was also given five units of pRBC and one unit of FFP. On hospital day 3, she began a daily dose of Venofer started until the day of discharge. Her hemoglobin and hematocrit stabilized to 9.5/28 in a matter of 3 days.

She was scheduled for an outpatient follow-up visit 4 days post-discharge to be scheduled for monthly Lupron injections and definitive surgical management via abdominal myomectomy; however, she was unable to be contacted despite more than ten voicemails and emails being left and the absence of family members.

## Discussion and conclusion

In AUB-L, several mechanisms are thought to cause bleeding. Fibroids are thought to be monoclonal tumors arising from the myometrium [5]. These tumors regulate the transforming growth factor-beta (TGF-β) pathway, which in turn affects the endometrial vascular system [6]. Fibroids may cause dysregulation of vasoactive growth factors overcoming platelet action, increased endometrial surface area, increased myometrial rigidity, ulceration, or compressive pressure [7]. Immunohistochemistry (IHC) and flow cytometry studies demonstrate that fibroblasts form a major proportion of the cells in fibroid tissues [5]. Genetic factors influencing uterine fibroid growth include the mutations *MED12*, *HMGA2*, and *COL4A5-A6*, which trigger downstream expression of factors implicated in angiogenesis, including Insulin-like growth factor IGF-1 and IGF-2 [8].

Uterine fibroids are a common cause of AUB-L, found in 25–50% of women with abnormal uterine bleeding [9]. Incidence of uterine fibroids peaks in the fifth decade of age [10]. Exact global prevalence is unknown due to limited population-level data and variations in classification across jurisdictions. A population-based study from 1995 in the USA found a cumulative incidence of fibroids above 66% via ultrasound of women approaching 50 years [11]. However, ultrasound is highly sensitive and not all detected fibroids are clinically significant.

Fibroids grow at variable rates prior to menopause but are usually slow-growing. In a longitudinal study, average fibroid growth was only 0.5 cm per year in diameter, but growth of 3 cm per year in diameter or greater was observed [12]. Fibroid growth may lead to abnormal uterine bleeding, and to depletion of the body’s iron stores, explaining the IDA and severely low hemoglobin, low hematocrit, and low MCV seen in our patient.

Physiological compensation for anemia may include increased cardiac output, enhanced oxygen unloading from the Hb by a shift to the right of the Hb–O2 dissociation curve, or increased Erythropoietin (EPO) production [13]. Documented increases in cardiac output in patients with anemia maintain tissue perfusion by activating the renin–angiotensin–aldosterone system to retain water and sodium and increase blood volume [14]. The tolerable limit of anemia, that is, the threshold hemoglobin value below which patients develop ischemic organ dysfunction and cardiac failure, is not known definitively. Further, there is little consensus on a specific hemoglobin or hematocrit value to use as indication for transfusion. The 10/30 rule, that transfusion is necessary when hemoglobin falls below 10 g/dL or hematocrit below 30%, is outdated [15]. Transfusion of one unit of RBC increases hematocrit by approximately 3% and hemoglobin concentration by 1 g/dL in a 70-kg non-bleeding adult [16].

Similar cases of severely low hemoglobin caused by abnormal uterine bleeding are rare. Three relevant cases are briefly reviewed here. In 2013, a 21-year-old woman with a history of abnormal uterine bleeding and altered mental status reported a hemoglobin level of 1.7 g/dL [17]. In 2021, a 42-year-old Hispanic woman reported a hemoglobin level of 1.4 g/dL. Her bleeding was similarly caused by leiomyomas; however, she was non-ambulatory [18]. In 2005, a 29-year-old woman had a hemoglobin of 1.7 g/dL concurrent with bulimia and celiac disease. She required assistance with ambulation and had severe symptoms [19]. All three cases of nulliparous women with hemoglobin of 1.7, 1.4, and 1.7 g/dL, respectively, were resolved by surgical intervention instead of embolization or hormonal therapy. It has been reported that embolization has a better short-term prognosis, but total hysterectomy leads to better long-term outcome; fertility, of course, is sacrificed [20].

Extremely severe hemodilution was reported in a 45-year-old Japanese man with a hemoglobin of 0.6 g/dL during liver transplantation surgery that caused massive bleeding [21]. To the author’s knowledge, this reading is the lowest hemoglobin ever recorded. The anomalous result led researchers to investigate the sensitivity of their measuring instruments. Testing using diluted samples of blood revealed that it is accurate to within 0.15 g/dL of the 0.6 g/dL result [21].

To date and to the best of the author’s knowledge, only six cases of severe anemia caused by abnormal uterine bleeding with hemoglobin levels < 2.0 g/dl have been reported in the medical literature [17–19, 22]. A 2006 study attempting to quantify the total direct cost to the US healthcare system of uterine leiomyomas estimated the burden to exceed $2 billion USD [23]. The present case adds to these other case reports of patient survival with a hemoglobin level of < 2 g/dL.

## Data Availability

Supporting data can be requested from the corresponding author of this paper.

## References

[1] Davis E, Sparzak PB. Abnormal uterine bleeding. [Updated 2023 Sep 4]. In: StatPearls. Treasure Island (FL): StatPearls Publishing; 2024 Jan-. https://www.ncbi.nlm.nih.gov/books/NBK532913/30422508

[2] Barros VV, Hase EA, Salazar CC, Igai AMK, Orsi FA, Margarido PFR (2022). Abnormal uterine bleeding and chronic iron deficiency. Revista brasileira de ginecologia e obstetrician.

[3] Munro MG, Critchley HOD, Fraser IS, Menstrual Disorders Committee FIGO (2018). The two FIGO systems for normal and abnormal uterine bleeding symptoms and classification of causes of abnormal uterine bleeding in the reproductive years: 2018 revisions. Int J Gynaecol Obstet.

[4] Bradley LD, Gueye NA (2016). The medical management of abnormal uterine bleeding in reproductive-aged women. Am J Obstet Gynecol.

[5] Holdsworth-Carson SJ, Zaitseva M, Vollenhoven BJ, Rogers PA (2014). Clonality of smooth muscle and fibroblast cell populations isolated from human fibroid and myometrial tissues. Mol Hum Reprod.

[6] Navarro A, Bariani MV, Yang Q, Al-Hendy A (2021). Understanding the impact of uterine fibroids on human endometrium function. Front Cell Dev Biol.

[7] Stewart EA, Nowak RA (1996). Leiomyoma-related bleeding: a classic hypothesis updated for the molecular era. Hum Reprod Update.

[8] Baranov VS, Osinovskaya NS, Yarmolinskaya MI (2019). Pathogenomics of uterine fibroids development. Int J Mol Sci.

[9] Don EE, Middelkoop MA, Hehenkamp WJK, Mijatovic V, Griffioen AW, Huirne JAF (2023). Endometrial angiogenesis of abnormal uterine bleeding and infertility in patients with uterine fibroids-a systematic review. Int J Mol Sci.

[10] Kulkarni MR, Dutta I, Dutta DK (2016). Clinicopathological study of uterine leiomyomas: a multicentric study in rural population. J Obstet Gynaecol Ind.

[11] Velebil P, Wingo PA, Xia Z, Wilcox LS, Peterson HB (1995). Rate of hospitalization for gynecologic disorders among reproductive-age women in the United States. Obstet Gynecol.

[12] DeWaay DJ, Syrop CH, Nygaard IE, Davis WA, Van Voorhis BJ (2002). Natural history of uterine polyps and leiomyomata. Obstet Gynecol.

[13] Metivier F, Marchais SJ, Guerin AP, Pannier B, London GM (2000). Pathophysiology of anaemia: focus on the heart and blood vessels. Nephrol Dialysis Transplant.

[14] Fountain JH, Kaur J, Lappin SL, Fountain JH (2023). Physiology, renin angiotensin system. StatPearls.

[15] Carson JL, Stanworth SJ, Roubinian N, Fergusson DA, Triulzi D, Doree C, Hebert PC (2016). Transfusion thresholds and other strategies for guiding allogeneic red blood cell transfusion. Cochrane Database Syst Rev.

[16] Liumbruno G, Bennardello F, Lattanzio A, Piccoli P, Rossetti G (2009). Recommendations for the transfusion of red blood cells. Blood Transfus.

[17] Çan Ç, Gulacti U, Kurtoglu E (2013). An extremely low hemoglobin level due to menorrhagia and iron deficiency anemia in a patient with mental retardation. Int Med J.

[18] Chai AL, Huang OY, Rakočević R, Chung P (2021). Critical iron deficiency anemia with record low hemoglobin: a case report. J Med Case Reports.

[19] Jost PJ, Stengel SM, Huber W, Sarbia M, Peschel C, Duyster J (2005). Very severe iron-deficiency anemia in a patient with celiac disease and bulimia nervosa: a case report. Int J Hematol.

[20] Psilopatis I, Fleckenstein FN, Collettini F, Can E, Frisch A, Gebauer B, Fehrenbach U, Torsello GF, Schnapauff D, David M, Wieners G (2022). Short- and long-term evaluation of disease-specific symptoms and quality of life following uterine artery embolization of fibroids. Insights Imaging.

[21] Kariya T, Ito N, Kitamura T, Yamada Y (2015). Recovery from extreme hemodilution (hemoglobin Level of 0.6 g/dL) in cadaveric liver transplantation. A A Case Rep.

[22] Kawano M, Okamoto M, Yano M, Kawano Y (2022). Life-threatening anemia due to uterine fibroids: a case series. Exp Ther Med.

[23] Mauskopf J, Flynn M, Thieda P, Spalding J, Duchane J (2005). The economic impact of uterine fibroids in the United States: a summary of published estimates. J Women Health.

